# Light chain deposition disease: pathogenesis, clinical characteristics and treatment strategies

**DOI:** 10.1007/s00277-024-05911-9

**Published:** 2024-08-28

**Authors:** Raffaella Cassano Cassano, Angelo Giovanni Bonadio, Maria Livia Del Giudice, Domenico Giannese, Sara Galimberti, Gabriele Buda

**Affiliations:** 1 Department of Clinical and Experimental Medicine, Hematology, University of Pisa, 56126 Pisa, Italy; 2 Department of Surgical, Medical, Molecular Pathology and Critical Area, Division of Surgical Pathology II, University of Pisa, Via Paradisa 2, 56124 Pisa, Italy; 3 Department of Clinical and Experimental Medicine, Nephrology Unit, University of Pisa, 56126 Pisa, Italy

**Keywords:** Light chain deposition disease, Plasma cells, Autologus stem cells transplant, LCDD, MGRS, Monoclonal gammopathy

## Abstract

Light chain deposition disease (LCDD) is a rare hematologic disorder characterized by the deposition of non-amyloid monoclonal light chains in several organs. Together with renal impairment is being the primary morbidity associated with this disease. Due to its rarity, randomized clinical trials lack to explore treatment strategies and there are no approved or universally accepted standard of care treatment options. We aimed to provide a systematic summary of histological and clinical aspects of LCDD and treatment options of available literature therapies strategies. Currently, drugs used to treat multiple myeloma are recommended when LCDD patients also presented multiple myeloma. Anyway, in patients with LCDD that is not associated to multiple myeloma, haematopoietic stem cell transplantation (ASCT) and chemotherapy with thalidomide, dexamethasone, bortezomib are also recommended. In eligible patients, bortezomib-based chemotherapy followed by ASCT appears to be an effective treatment option with durable hematologic remission and organ responses. Although it appears that the patients undergoing ASCT seem to achieve deeper and durable hematologic remissions and organ responses, no statistically significant superiority can be demonstrated over non-transplant or standard chemotherapy-based approaches. As retrieved by our review, bortezomib-based therapy appears to be favorable strategy as long as no dose modification is required for renal impairment, and early hematologic responses as a recovery of renal function. Encouraging data were also demonstrated by treatment lenalidomide or melpalan based. Moreover, new myeloma treatment strategies, as monoclonal antibody Daratumumab, seem to be effective in LCDD. Instead, renal allograft is not recommended, due to high incidence of relapse.

## Introduction

Light Chains Deposition Disease (LCDD) is a clonal plasma cell disease characterized by the deposition of non-amyloid monoclonal light chains, kappa, more frequently, or lambda in several organs. It is a relatively rare condition with unknown incidence in literature because often its not diagnosed, in asymptomatic phases. The median age of patients at diagnosis is approximately 58 years, with a slightly higher preponderance for men [1].

According to the 5th edition of 2022 World Health Organization (WHO) classification of tumors of hematopoietic and lymphoid tissues [2], LCDD is categorized as a Monoclonal immunoglobulin deposition disease (MIDD), a multi-system group of disorders characterized by the deposition of abnormally truncated monoclonal Ig molecules in various organs, first of all in kidney, where accumulation of monoclonal immunoglobulin molecules involves vascular basement membranes, glomerular basement membranes (GBMs), and tubular basement membranes (TBMs) without a fibrillary, crystalline, or microtubular appearance on electron microscopy. The deposition is typically represented by negative to red Congo staining, a single light chain isotype or single heavy chain subclass in immunofluorescence, and a powdery deposition on electron microscopy. MIDD includes light chain deposition disease (LCDD), the most prevalent disorder that constitutes approximately 80% of MIDD, heavy chain deposition disease (HCDD) and light and heavy chain deposition disease (LHCDD), considered extremely rare [3, 4].

As LCs are overproduced by an abnormal clone of B cells, LCDD is usually described in the course of plasma cell dyscrasias, not only multiple myeloma (11–65%) but also monoclonal gammopathy of undetermined significance (MGUS) (32–86%), or macroglobulinemia (2%), or other lymphoproliferative disorders (2–3%). [5].

MGUS is considered to be a premalignant condition characterized by the presence of monoclonal gammopathy (< 3 g/dL) without clinical features or organ damage. Therefore, a new definition "Monoclonal Gammopathy of Renal.

Significance (MGRS)" has recently been proposed for MGUS with concurrent renal disease (Table 1). More recently, International Kidney and Monoclonal Gammopathy Research Group Consensus characterized LCDD as part of monoclonal gammopathy of renal significance (MGRS), a broader spectrum of B-cell proliferative disorders known to produce monoclonal immunoglobulins toxic to kidneys [3, 6]. Morover, it could occur in absence of any detectable hematological disorder and this pathological condition is called idiopathic LCDD.

**Table 1:** Spectrum of clinical presentation of MGRS [6]

Monoclonal immunoglobulin deposits	Organized	a) Fibrillar	Immunoglobulin related amyloidosis Monoclonal fibrillary glomerulonephritis
		b) Microtubular	Immunotactoid glomerulonephritis Cryoglobulinaemic glomerulonephritis type I and type II
		c) Inclusions or crystalline deposits	Light-chain proximal tubulopathy Crystal storing histiocytosis (Cryo) crystalglobulin glomerulonephritis
	Non-organized		Monoclonal immunoglobulin deposition disease Proliferative glomerulonephritis and monoclonal immunoglobulin deposits. Miscellaneous
No monoclonal immunoglobulin deposits			C3 glomerulopathy with monoclonal gammopathy Thrombotic microangiopathy

This review aims to provide a systematic summary of histological and clinical aspects of LCDD and treatment options of available literature therapies strategies.

## Pathogenesis and histological diagnosis

The clinical presentation of LCDD is extremely heterogeneous, due to the clinical symptoms depend on which tissues are affected by light chain (LC) deposition and consequently by the organ dysfunction, but the kidney involvement is always demonstrated. Renal involvement and disfunction usually dominate clinal aspects and disease course, however hepatic, cardiac and neural deposits have also been documented. Rare cases of LCDD without glomerular findings have also been reported. [5].

Because free light chains (FLCs) are rapidly cleared from the serum and are largely filtered by the glomeruli, reabsorbed in proximal tubules by receptor mediated endocytosis, and degraded by lysosmal enzymes in tubular cells, the kidney is a prominent target for LC deposition and it is often damaged. Bence Jonce Protein detected in the urine is evidently a spillover of LC that escaped reabsorption and metabolism by renal tubules. However, morphological renal lesions do not seem to correlate with patient survival in LCDD. [5].

Renal symptoms, primarily proteinuria or neprhotic syndrome, often dominate clinical presentation due to monoclonal toxicity to renal tissues. Moreover, the light chain in LCDD exhibits somatic mutations in the L12a gene, that may change the LC structure, leading to abnormal deposition or nephrotoxicity. In absence of therapy, the clinical course is progressive chronic kidney disease (CKD) or acute kidney injury (AKI), requiring renal replacement therapy.

Glomerular-filtered FLCs are reabsorbed in the mesangium or proximal tubules. Mesangial cells (MCs) secrete extracellular matrix (ECM), mediators and enzymes such as matrix metalloproteinases (MMPs) to support and maintain the glomerulus structure. The increasing deposition of ECM proteins and monotypic LCs results in mesangial nodularity within the glomerulus. MCs are critical in the pathogenesis of glomerulosclerosis because FLCs bind to putative receptors residing in caveolae on the plasma membrane of MCs to activate intracellular signaling that leads to the overexpression of the receptor. The majority of monoclonal LCs in LCDD are kappa that has a particularly long CDR1 loop which may promote conformational changes or the aggregation of the FLCs through its multiple hydrophobic residues. [7].

The complementarity-determining region (CDR) of LCDD-associated FLCs has unusual hydrophobic amino acids (AA) substitutions, and κ-LCs in LCDD have an exposed b-edge that is part of the antigen binding site in the CDR2 loop, whereas λ-LCs do not. This exposed edge leads to spontaneous aggregation of the k-LCs into oligomers, which may form granular deposits. FLCs inhibit the release of MMP-7 from MCs, which degrades tenascin-C, resulting in increased ECM. Ribosomal S6 kinase (RSK) can phosphorylate a variety of transcription factors, including c-fos, promoting nuclear signal transduction: the activation of c-fos results in the transcription of platelet derived growth factor (PDGF)-β, that induces human fibroblast cell membrane wrinkling in MCs leading to further increase interactions with FLCs. Nuclear factor kappa-light chain-enhancer of activated B cells (NF-κB) and c-fos are induced to migrate to the nucleus by LCDD-associated FLCs. The activation of the transcription factor NF-κB plays an important role in interleukin-1 (IL-1)-induced monocyte chemoattractant protein-1 (MCP-1) expression. NF-κB translocates into the nucleus and binds to specific DNA sequences on NF-κB response genes, such as MCP1, regulated upon activation normal T-expressed and secreted (RANTES), and ICAM-1, resulting in enhanced transcription and generation. Moreover, Kon and colleagues have shown a functional interaction between NF-κb and SMAD, two early-intermediate transcription factors, to activate COL7A1 expression, an ECM-related gene. When MCs are exposed to FLCs in LCDD, transforming growth factor (TGF)-β production is increased. TGF-β inhibits mesangial proliferation and increases ECM secretion, including tenascin. Cast formation can be seen in as many as one-third of LCDD cases. Tubulointerstitial inflammation and fibrosis are the main features of cast formation, with hard and often fractured protein deposits in distal renal tubules (casts), composed of uromodulin and FLCs. Moreover, glomerular capillary walls have deposits of FLCs [8, 9].

In renal biopsies, characteristics granular electron dense deposits along basement membranes were retrieved by electron microscopy, between the lamina densa and subendothelial space, or in the TBMs, in the arterioles' intima and BMs, interstitial capillary BMs. Immunoelectron microscopy of kappa or lambda chains is useful when the deposits cannot be detected by immunofluorescence or electron microscopy.

**Fig. 1:**
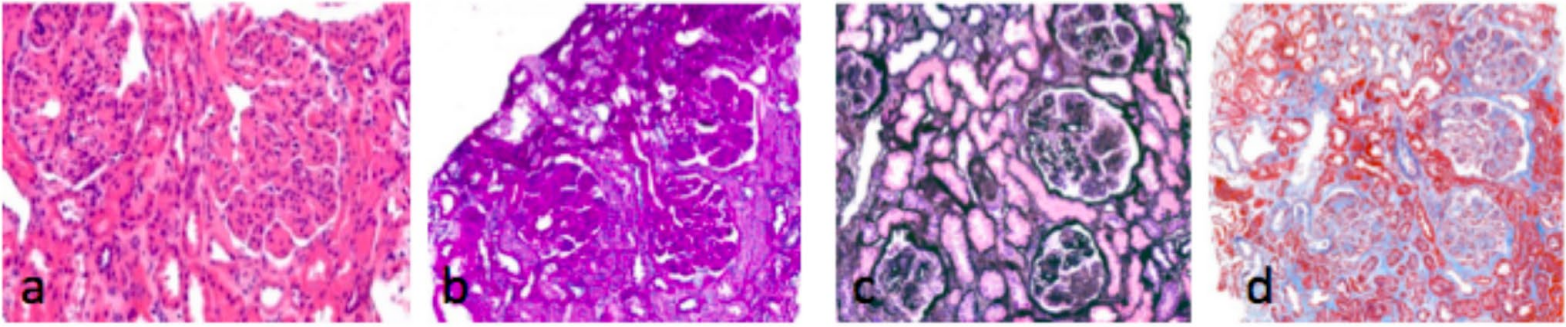
Monoclonal immunoglobulin deposition disease: **a**) stained with Hematoxylin–Eosin 20x, **b**) PAS, **c**) Jones Methenamine, **d**) Masson Trichrome, 10x. Nodular sclerosing light are present in the glomerular mesangial regions. The nodules typically have an acellular eosinophilic center with residual cells at the periphery. The nodules are very PAS positive, less JMS positive than the nodules of diabetic glomerulosclerosis, MTS positive and Congo-red stain negative notshown here

Light microscopy, instead, shows nodular mesangial sclerosis, tubular atrophy and interstitial fibrosis and increased lobulation and duplication of GBM, which shows endocapillary proliferation and membreano-proliferative glomerulonephrtis lesions, prominently in the glomeruli. (Fig. 1) Rarely, linear light chain deposits along the TMBs are present in LCDD, without glomerular lesions. The deposits of light chains are typically negative to Rosso Congo red staining. The diagnostic immunohistological finding of LCDD is the exclusive deposition of light chain isotype, kappa or lambda, without heavy chain in GMBs and TMBs [8, 10, 11].

**Fig. 2:**
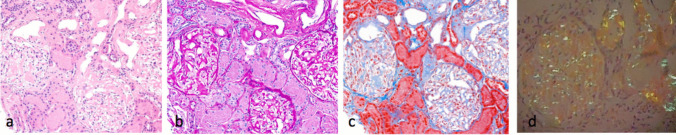
Amyloidosis in hematoxylin–eosin (**a**), PAS stain (**b**), Masson trichrome stain and Congo-Red stain (**c**), × 10; Red-Congo stain shows apple green birefringence at polarized microscopy (**d**). Amyloid deposits in the mesangial site with the formation of nodular areas and in the walls of the vessels. The amyloid deposits have a paler appearance than sclerosis of diabetic glomerulosclerosis and light chain deposition disease

**Fig. 3:**
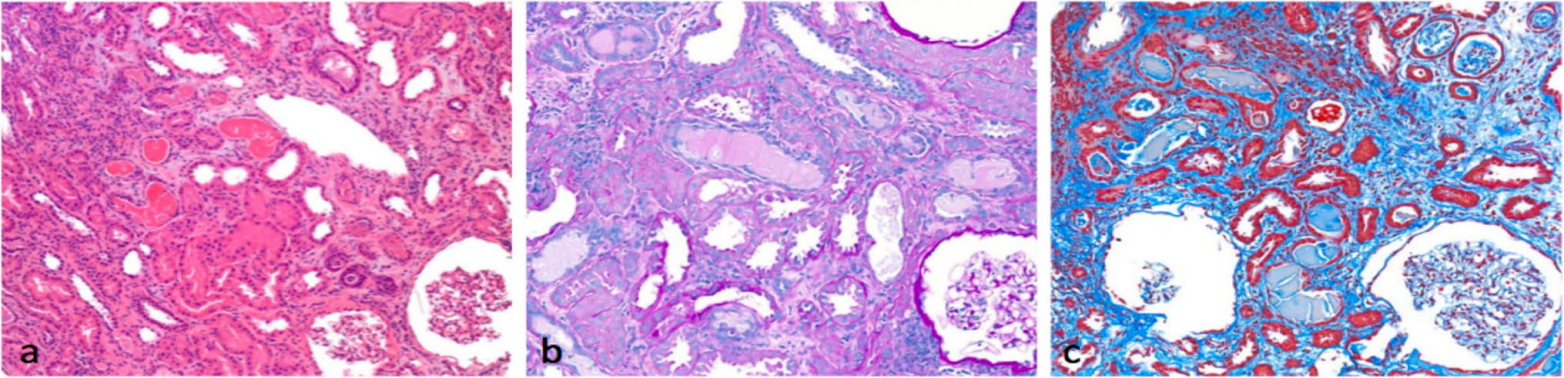
Myeloma cast nephropathy; hematoxylin–eosin (**a**), PAS stain (**b**) and Masson trichrome stain (**c**), × 10: large refractile, hyaline casts in the dilatated renal cortical tubules. The casts are dense, strongly eosinophilic, acellular and may appear multilamellar. With the trichrome stain, the casts appear polychromatic, with a mixed red-blue coloration

Renal diseases with deposition of monoclonal immunoglobulins or their components include not only LCDD, but also AL amyloidosis (Fig. 2), myeloma cast nephropathy (Fig. 3), light chain proximal crystal tubulopathy (light chain associated Fanconi's syndrome), type 1 cryoglobulinemia, and crystal storing histiocitosis. They could be differentiated by depositions distribution, if in glomeruli, arteries or in tubuloniterstritium. For example, cast nephropathy, light chain associated Fanconi's syndrome, and crystal storing histiocytosis are included in tubulointerstitium category. [11, 12].

Approximately 35% of patients with LCDD have extra renal lesions, that occur in the hepatic sinusoids, choroid plexus or myocardium [13].

In the endomyocardial biopsies, analyzed by electron microscopy, are observed amorphous nonfibrillar deposits around several small vessels including capillaries and small arteries, which were consistent with light-chain deposits. CIDD (Cardiac nonamyloidotic immunoglobulin deposition disease) is defined as the presence of immunofluorescent Ig deposition in the cardiac interstitium or vascular walls in the absence of amyloid as evaluated by Congo Red, Thioflavin T and EM examination [14, 15].

At liver biopsy, massive infiltration of a homogeneous and dense eosinophilic material are usually found to occupy the perisinusoidal space in LCDD patients; immunostaining demonstrates diffuse light chain kappa or lambda restriction, and Congo red staining is negative.

## Clinical presentation

Clinical manifestations depend on which organs are involved. LCDD can present as kidney insufficiency, hyperthension, proteinuria, microscopic hematuria, more rarely renal tubular acidosis and nephrotic syndrome. Albuminuria levels do not correlate with the presence of glomerulonephritis and can be present even without glomerulosclerosis. Kidney function declines rapidly as rapidly progressive glomerulonephritis or acute tubulointerstitial nephritis and early loss of function that may occur even within few weeks or months, causing rapidly progressive AKI.

CKD at the diagnosis has a significant impact of renal survival with those who had CKD stage 2–3 at diagnosis remain dialysis independent for median 9 years compared with only 2.7 years among cohort with CKD stage 4–5.

There was no significant difference in survival between patients who had nephrotic range proteinuria, ie > 3 g/24 h, at diagnosis and those with subnephrotic range proteinuria, even though proteinuria > 6 g/24 h is associated with reduced renal survival.

Moreover, it's possible to affirm that survival of LCDD patients is not influenced by the entity of bone marrow plasmacell infiltrate. Anyway, combined LCDD and myeloma cast nephropathy exhibits worsen prognosis and clinical features that more closely resemble those in myeloma.

Interestingly, in some cases, the M-spike cannot be detected on serum or urine protein electrophoresis or immunofixation on urine or blood.

Extrarenal LCDD most commonly involves the liver but may affect organs with a high degree of vascularity. Cardiac LCDD has been noted, cardiological symptoms could be heterogeneous manifesting in incidents of arhythmias and even as an atrial mass. Although nonamyloidotic immunoglobulin light chain deposits in the myocardium differ in distribution and ultrastructural organization from the fibrillar deposits of AL disease, an analogous pattern of diastolic dysfunction and conduction disturbances results as arhytmias or congestive heart failure. The diagnosis should be considered in patients with a plasmacytic dyscrasia and restrictive cardiomyopathy in whom Congo red staining of endomyocardial biopsy tissue is negative [13–15].

Patients with pulmonary LCDD present with characteristics of nodules, cysts, and ancillary findings at HRTC and there was no zonal predominance of either cysts or nodules, dyspnea, hemoptysis and chest pain, though symptomatic cases are rare; more typically pulmonary LCDD is an incidental finding in asymptomatic patients with lymphoproliferative disorders. [16] The disease appeared stable in the majority of cases with no serial change in HRCT appearances.

Liver involvement may be asymptomatic of manifest with mild to moderate transaminase elevation, portal hypertension of fulminant liver failure [17, 18].

Therefore, the initial evaluation of all the patients should include a detailed history and physical examination along with a comprehensive laboratory evaluation, which included complete blood counts, serum electrolytes, creatinine, 24-h creatinine clearance, serum and urine protein electrophoresis with immunofixation studies. All the patients should undergo to a bone marrow aspirate and biopsy, skeletal surveys and skull, spine and pelvis magnetic resonance imaging (MRI). Electrocardiogram and transthoracic echocardiography to evaluate cardiac function should be also carried out.

Hematological response to treatment, as polyclonal retention of FLCs associated with CKD, should be evaluate after 6 months at least from the start of chemotherapy and evaluating the difference between pathogenic and non pathogenic light chain concentration (dFLC). CR is defined as normalization of FLC ratio in the absence of detectable monoclonal protein by serum and urine immunofixation electrophoresis, hematologic very good partial response (VGPR) as a decrease if dFLC to < 40 mg/L, hematologic partial response (PR) as > 50% decrease if dFLC from baseline, no response as < 50% decrease in dFL from baseline. [19, 20].

Renal response is considered decreasing of 50% in 24 h urine protein concentration and < 25% decline in renal function. [21–24] (Table 2).

**Table 2:** Evaluation of Hematological response

Complete Response (CR)	normalization of FLC ratio in the absence of detectable monoclonal protein by serum and urine immunofixation electrophoresis
Very Good Partial Response (VGPR)	decrease if dFLC to < 40 mg/L
Partial Response (PR)	> 50% decrease if dFLC from baseline
No Response (NR)	< 50% decrease in dFLC from baseline

## Treatment options

Due to the rarity of the disease and lack of randomized clinical trials, there are currently no Food and Drug Administration (FDA) or European Medical Association (EMA) approved therapies or universally accepted standard of care treatment options available for LCDD. The main goal of treatment is to slow the production and tissues deposition of light chains to prevent further damage and organ disfunction.

During the past, most common strategies to treat myeloma were represented by alkylating agents; as in multiple myeloma those therapies were proposed also for LCDD. Concerning to treatment based on alkylating agents, Pozzi et al. compared the efficacy of alkylating (ie. doxorubicin) plus corticosteroids and VAD/VAMP demonstrating neither was significantly associated with improvement patients survival. However, VAD/VAMP seems to show a non significant trend towards a better patients prognosis. According to Pozzi et al., the antifibrotic action of high dose corticosteroids could represents a possible explanation[9].

The introduction of proteasome inhibitor in therapeutic armamentarium revolutionized the treatment of plasma cell dyscrasias involving the kidney. In that background, in eligible patients, many physicians proposed autologous stem cell transplant preceded by a bortezomib based induction [25–29]. In the monocentric experience of Princess Margaret Hospital, in Toronto, Canada, Bortezomib plus Dexamethasone induction regimen to ASCT has been compared to Dexamethasone in six patients affected by LCDD. [9] Conditioning regimen performed was Melphalan, dose adjusted to clinical condition as per physician discretion. After induction therapy, median 4 cycle in BorD arm and 3 to 6 cycle in Dexamethasone arm, four out six patients achieved a PR, and 2/6 attained a SD. The study reported hematologic and renal responses (based on serum creatinine and 24-h proteinuria) post-induction and 6 months post-ASCT.

At day 100, post ASCT overall response rate was 100%, 83% patients achieved a CR and one PR, all with clinical benefit. At 6 months after ASCT, all of the patients achieved a renal response post-transplant manifested mainly by decrease of proteinuria > 50% and two-thirds of the patients achieved CR, 17% had a PR, and 17% had no response.

BD arm showed a median time of kidney response of 3 months vs 6 months in dexamethasone group. Dialysis free survival at 2 years was 100%. [30, 31].

Kastritis et al., in 2009 reported four patients from a single center who received BorD for induction chemotherapy prior to ASCT. Two out of four patients achieved hematologic CR. Three (75%) patients subsequently underwent ASCT. All patients achieved a renal response, and all patients were alive at the time of the last follow-up (range: 10—18 months). [32]. Based on this case series, bortezomib-based regimens seemed to be a reasonable option for patients prior to ASCT.

In Italian experience of Padua group, a second ASCT in a patient who achieved a PR has been performed, however obtaining, also after the second autologous, a PR. [33].

Bortezomib may have a protective role on renal parenchyma due to inhibition of NF-kB activity, reduction of TGF-beta1 levels and down regulation collagen production, improving glomerular function and reducing proteinuria. The monoclonal light chains interact, in fact, with mesangial cells receptor, initiating a cascade activation pathway that include NF-kB, stimulating cytokines production and inflammatory reaction. PDGF-beta and TGF-beta are also induced by light chain in the mesangial cell interaction in LCDD, causing proliferation and activation of collagen production genes, responsible of glomeruloscleloris. [34–41].

In relapsed patients, Bortezomib seems to be effective also in relapsed after alkylating agents, as demonstrated in a cohort 4 patients enrolled in a Greek study (Kastritis et al.), one previously exposed to VAD (vincristine, doxorubicine and dexamethasone) and one to cyclophosfamide plus prednisone; the remaining two were previously untreated. [32]

All study cohort presented with impaired renal function, non selective proteinuria and poorly controlled hypertension, serum free light chains elevated, with abnormal kappa/lambda ratio, one patient presented also echocardiographic evidence of heart involvement. After 6 cycles of induction with Bortezomib and dexamethasone, 2 patients achieved a CR (one of them was the VAD refractory patient), with a normal FLC ratio, while the other 2 had > 50% decrease of the involved light chain, all achieved adequate hypertension control, with less than half of the drugs they used before. As expected, the toxicity was manageable and was represented mostly by neuropathy, constipation, transient orthostatic hypotension and transient elevation of liver enzymes. All patient underwent to ASCT achieved a CR with only trace proteinuria. Patient with heart involvement improved his diastolic dysfunction and reduction of 1 mm the interventricular septum thickness. After a follow up of 10–18 months, all patients are alive but one patient, who wasn't candidate to ASCT, proteinuria recurred two months after interruption of VD, although criteria for hematological relapse were not fulfilled.

Comparing proteasome inhibitor to immunomodulatory drugs among individual studies, few are remarkable such as Sayed et al., 2015 who reported the largest single-center retrospective analysis of idiopathic LCDD from the United Kingdom National Amyloidosis Center. Out of the 25 evaluable patients, four underwent ASCT and achieved hematologic CR. Similarly, 89% of the patients in the bortezomib group (*n* = 8/9) and 27% of the patients in the thalidomide group (*n* = 3/11) achieved CR. Only one patient received lenalidomide and had a PR. [23]

Lorenz et al. proposed ASCT after different induction regimens in a cohort of six patients affected by LCDD, whom one patient was on hemodialysis, another patient had a cardiac and liver involvement. Prior to bone marrow transplant, four patients received dexamethasone only for 3 months, one patient a combination of thalidomide and dexamethasone for 4 months and one no chemotherapy. One patient died due to multi-systemic organ failure 26 days after ASCT but had diffuse systemic organ involvement of LCDD. That suggests patients with extrarenal manifestations of LCDD, i.e. cardiac deposition, may be at higher risk wih ASCT. All the remain five achieved a hematological response after ASCT although two ultimately relapsed and required further chemotherapy, one after 7 months, the second patient after 28 months and they received lenalidomide based treatment. All patients achieved a renal response characterized by decreasing of 50% in 24 h urine protein concentration and < 25% decline in renal function. [25]

With the advent of second generation immunomodulatory imide drugs (IMiDs), growing interest has been shown towards the Lenalidomide [42].

In a cases series of Kimura et al. they explore lenalidomide-based therapy with developing renal impairment associated with nephrotic syndrome; one patient received 6 cycles of bortezomib plus lenalidomide plus dexamethasone followed by lenalidomide plus dexamethasone, one patient was treated for 30 months by the combination lenalidomide plus dexamethasone and the last one underwent to autotransplant after 4 cycle of lenalidomide plus dexamethasone followed by low-dose lenalidomide maintenance therapy. [43] In all of the reported cases, lenalidomide-based therapy improved the renal impairment by decreasing the high levels of serum monoclonal light chains, resulting in a marked reduction in proteinuria, also in a bortezomib refractory patient. Lenalidomide is known to stimulate T and natural killer cells for MM cells, which produce cytokines such as interleukin-6, tumor necrosis factor-α, transforming growth factor-β (TGF-β) and vascular endothelial growth factor (VEGF) mediated via the NF-κB pathway. The interaction of renal glomerular mesangial cells with monoclonal light chains has been shown to activate cytokines such as TGF-β and VEGF in combination with the high production of matrix and extracellular matrix proteins, which compose the glomerular lesions in LCDD. Interestingly, lenalidomide has been also shown to down-regulate the production of cytokines that include TGF-β by activated monocytes while simultaneously up-regulating IL-2 and interferon-γ production, which promotes the activation of T and natural killer cells. [43, 44]

Regarding to renal transplantation, many studies evaluated outcome of renal transplantation in LCDD patients. Kobayashi et al. in 2023 described a case report of a patient who relapsed after long-term remission following renal transplantation. At 2 years post-transplantation, a graft biopsy performed after complete remission was achieved, showing some glomeruli with residual nodular lesions similar to the pre-treatment renal biopsy findings. However, the enlarged subendothelial space disappeared. She remained in complete remission serologically for 6 years. Subsequently, the ratio of serum κ/λ-free light chains decreased gradually. She underwent a transplant biopsy approximately 12 years after renal transplantation due to increased proteinuria and decreased renal function. Compared with the previous graft biopsy, almost all glomeruli showed advanced nodules formation and subendothelial expansion. [45]

Leung N. et al. explored the efficacy of renal transplantation in LCDD patients. The authors retrospectively reviewed outcome of 7 patients with LCDD who underwent kidney transplantation, whom five patients were on dialysis before transplantation. They observed that LCDD recurred after a median of 33.3 months in 5 of the 7 patients. One patient remains on dialysis, whereas the other 4 died. One patient died of progression of hematological condition 3 months after kidney transplantation without evidence of recurrence. Only 1 patient remains recurrence free after 13 years with normal renal allograft function. The concluded that although long-term benefits are occasionally seen, renal allograft survival is reduced significantly in LCDD patients. [46] Kidney transplantation should not be an option for LCDD patients unless measures have been taken to reduce light chain production [47, 48].

Moreover, interesting data were demonstrated regarding to melphalan based treatment by Hiyamuta et al., 2015, who administered the combination of Bortezomib-Melphalan-Prednisone to a 70-year-old woman with multiple organ involvement (kidney, thyroid gland, heart and eyes) of LCDD. After the first chemotherapy cycle with bortezomib, cyclophosphamide and dexamethasone, hepatobiliary enzyme levels increased abruptly. A liver biopsy showed light chain deposition in Disse spaces. So, they decided to change treatment regimen, considering patient age and frailty. After two years of treatment with bortezomib, melphalan and prednisone (VMP) administered at shorter intervals of 6 weeks relative to regular cycles, the patient showed a hematological and organ response in a condition of acceptable tolerability. [49]

In a retrospective study of Heilman et al., nineteen patients with light-chain deposition disease were observed in a long-term patient and renal survival and the response to intermittent administration of melphalan and prednisone. All of the patients had some impairment of renal function at presentation, and 58% had a serum creatinine concentration greater than 4.0 mg/dL. Stabilization or improvement in renal function appear after chemotherapy in five of eight patients who had a serum creatinine concentration less than 4.0 mg/dL at the introduction of therapy. 82% of the 11 patients with a high serum creatinine concentration (greater than 4.0 mg/dL), progressed to end-stage renal disease despite therapy. Follow-up urine proteins demonstrated at least a 50% decrease in urine proteins excretion in five of 15 patients. [50]

In recent years, increasing attention is addressed to monoclonal antibody, as anti CD 38 approved for Multiple Myeloma. Daratumumab is an anti-CD38 monoclonal antibody that is highly effective in multiple myeloma patients as a single agent and in combination with proteasome inhibitors or immunomodulatory agents. Daratumumab was used in previously treated patients with AL amyloidosis with encouraging results. This agent became available in July 2017 in Italy for the treatment of relapsed/refractory multiple myeloma and is currently approved in the first line treatment of autologous transplant eligible patients and also in elderly ones.

Kastritis et al., in 2021 reported six LCDD patients who received 4-week consolidation course of daratumumab after 8 cycles of bortezomib, cyclophosphamide, and dexamethasone (VCd). All patients had renal involvement at the time of diagnosis and failed to achieve a CR after VCd. Three patients were already in hematologic VGPR, and the rest were in PR before daratumumab initiation. After four weekly daratumumab administrations, one patient improved to hematologic CR, while three remained in VGPR, and the rest of the two in PR. The free light chain (FLC) ratio normalized in 50% of patients after the consolidation as opposed to none prior. The interesting data were also about toxicities: only noticeable adverse event was mild injection-related reaction to daratumumab, and no hematological adverse event was reported [51]

## Conclusions

LCDD is a rare disease characterized by the deposition of non-amyloid monoclonal light chains, kappa, more frequently, or lambda in several organs. Due to its rarity, randomized clinical trials lack to explore treatment strategies and there are no approved therapies or universally accepted standard of care treatment options available for LCDD.

Currently, drugs used to treat multiple myeloma are recommended when LCDD patients also have multiple myeloma. Anyway, in patients with LCDD that is not accompanied by multiple myeloma, hematopoietic stem cell transplantation (HSCT) and chemotherapy with thalidomide, dexamethasone, bortezomib, lenalidomide, and alkylating drugs are also recommended. [52] First-line treatment strategies comprise systemic chemotherapy with or without ASCT to eliminate plasma cell burden in the bone marrow that produces light chains. This leads to long-term hematologic remission and improvement in organ function, evaluated by improvement in estimated glomerular filtration rate, serum creatinine, and proteinuria.

Although it appears that the patients undergoing ASCT seem to achieve deeper and more durable hematologic remissions and organ responses, no statistically significant superiority can be demonstrated over non-transplant or standard chemotherapy-based approaches. As retrieved by this review, bortezomib-based therapy appears to be favorable strategy as long as no dose modification is required for renal impairment, and early hematologic responses can be seen that reflect the recovery of renal function. A recent clinical trial of daratumumab in eight LCDD patients with a concomitant diagnosis of MM demonstrated reasonable hematologic and renal responses (50% achieved VGPR and 25% achieved renal response), which opens the possibility in further investigations into monoclonal antibodies [53].

There are several limitations associated with this systematic review. First, the mentioned studies explored in the analysis are retrospective and with a small number of patients. Furthermore, hematologic and organ responses could be different in each study in response evaluation criteria and in most of them follow-up remains short, making it difficult to collect meaningful conclusions about the long-term efficacy and toxicity. Moreover, in the absence of a diagnostic renal biopsy in some cases, it is difficult to distinguish whether renal impairment was due to LCDD versus other causes of MGRS. Larger retrospective analyses or randomized multicenter prospective studies with clearly defined hematologic and organ response evaluation criteria are needed to determine the efficacy and safety of the different treatment options available for this disease.

## Data Availability

1. Pozzi C, D'Amico M, Fogazzi GB, Curioni S, Ferrario F, Pasquali S, Quattrocchio G, et al. Light chain deposition disease with renal involvement: clinical characteristics and prognostic factors. Am J Kidney Dis. 2003; 2. Alaggio, R., Amador, C., Anagnostopoulos, I. et al. The 5th edition of the World Health Organization Classification of Haematolymphoid Tumours: Lymphoid Neoplasms. Leukemia 36, 1720–1748 (2022) 3. Menè P, De Alexandris L, Moioli A, Raffa S, Stoppacciaro A. Monoclonal Gammopathies of Renal Significance: Renal Biopsy and Beyond. Cancers (Basel). 2020 4. Ronco P, Plaisier E, Mougenot B, Aucouturier P. Immunoglobulin light (heavy)-chain deposition disease: from molecular medicine to pathophysiology-driven therapy. Clin J Am Soc Nephrol. 2006 Nov. 5. Kanzaki G, Okabayashi Y, Nagahama K, Ohashi R, Tsuboi N, Yokoo T, Shimizu A. Monoclonal Immunoglobulin Deposition Disease and Related Diseases. J Nippon Med Sch. 2019; 6. Wang Q, Jiang F, Xu G. The pathogenesis of renal injury and treatment in light chain deposition disease. J Transl Med. 2019 Nov 7. Leung N, Bridoux F, Nasr SH. Monoclonal Gammopathy of Renal Significance. N Engl J Med. 2021 8. Pozzi C, Locatelli F. Kidney and liver involvement in monoclonal light chain disorders. Seminars Nephrol. 2002 Jul 9. Ronco PM, Alyanakian MA, Mougenot B, Aucouturier P. Light chain deposition disease: a model of glomerulosclerosis defined at the molecular level. J Am Soc Nephrol. 2001 10. Lin J, Markowitz GS, Valeri AM, Kambham N, Sherman WH, Appel GB. Renal monoclonal immunoglobulin deposition disease: the disease spectrum. J Am Soc Nephrol. 2001 11. Herrera GA, Joseph L, Gu X, Hough A, Barlogie B. Renal pathologic spectrum in an autopsy series of patients with plasma cell dyscrasia. Arch Pathol Lab Med. 2004 12. Hudak M, Sardana R, Parwani AV, Mathewson RC, Gibson CG, Cohen PA, et al. Light chain deposition disease presenting as an atrial mass: a case report and review of literature. Cardiovasc Pathol. 2021 13. Fabbian F, Stabellini N, Sartori S, Tombesi P, Aleotti A, Bergami M. Light chain deposition disease presenting as paroxysmal atrial fibrillation: a case report. J Med Case Rep. 2007. 14. Koopman P, Van Dorpe J, Maes B, Dujardin K. Light chain deposition disease as a rare cause of restrictive cardiomyopathy. Acta Cardiol. 2009 Dec. 15. Colombat M, Gounant V, Mal H, Callard P, Milleron B. Light chain deposition disease involving the airways: diagnosis by fibreoptic bronchoscopy. Eur Respir J. 2007 16. Sweet DE, Wheeler CA, Kearns C, Marquis KM. Pulmonary Light-Chain Deposition Disease. Radiographics. 2022 17. Katzmann JA, Kyle RA, Benson J, Larson DR, Snyder MR, Lust JA. Screening panels for detection of monoclonal gammopathies. Clin Chem. 2009 18. Masai R, Wakui H, Togashi M, Maki N, Ohtani H, Komatsuda A. Clinicopathological features and prognosis in immunoglobulin light and heavy chain deposition disease. Clin Nephrol. 2009 19. Went P, Ascani S, Strøm E, Brorson SH, Musso M, Zinzani PL. Nodal marginal-zone lymphoma associated with monoclonal light-chain and heavy-chain deposition disease. Lancet Oncol. 2004 20. Katzmann JA, Clark RJ, Abraham RS, Bryant S, Lymp JF, Bradwell AR. Serum reference intervals and diagnostic ranges for free kappa and free lambda immunoglobulin light chains: relative sensitivity for detection of monoclonal light chains. Clin Chem. 2002 21. Ronco P, Plaisier E, Aucouturier P. Monoclonal immunoglobulin light and heavy chain deposition diseases: molecular models of common renal diseases. Contrib Nephrol. 2011. 22. Sayed RH, Wechalekar AD, Gilbertson JA, Bass P, Mahmood S, Sachchithanantham S, et al. Natural history and outcome of light chain deposition disease. Blood. 2015 23. Pozzi C, Locatelli F. Kidney and liver involvement in monoclonal light chain disorders. Semin Nephrol. 2002 Jul;22(4):319–30. PMID: 12118397. 24. Jimenez-Zepeda VH, Trudel S, Winter A, Reece DE, Chen C, Kukreti V. Autologous stem cell transplant for light chain deposition disease: incorporating bortezomib to the induction therapy. Am J Hematol. 2012 Aug;87(8):822–3. doi:https://doi.org/10.1002/ajh.23235. Epub 2012 May 28. PMID: 22641434. 25. Jimenez Zepeda VFN, Winter A, Reece D, Trudel S, Chen C, Rabea A, et al. Light chain deposition disease: impact of stem cell transplant on Hematological response achievement. 2010. 26. Kastritis E, Migkou M, Gavriatopoulou M, Zirogiannis P, Hadjikonstantinou V, Dimopoulos MA. Treatment of light chain deposition disease with bortezomib and dexamethasone. Haematologica. 2009 27. Lessi F., Castelli M, Trentin L, Altinier S, Piazza F, Adami F. Bortezomib-Dexamethasone As Induction Therapy for Light Chain Deposition Disease (LCDD): A Single Center Experience. Blood. 2012 28. Gharwan H, Truica CI. Bortezomib-based chemotherapy for light chain deposition disease presenting as acute renal failure. Med Oncol. 2012 29. Gertz MA. Managing light chain deposition disease. Leuk Lymphoma. 2012 30. Tovar N, Cibeira MT, Rosiñol L, Solé M, de Larrea CF, Escoda L. Bortezomib/dexamethasone followed by autologous stem cell transplantation as front line treatment for light-chain deposition disease. Eur J Haematol. 2012 31. Fineschi S, Reith W, Guerne PA, Dayer JM, Chizzolini C. Proteasome blockade exerts an antifibrotic activity by coordinately down-regulating type I collagen and tissue inhibitor of metalloproteinase-1 and up-regulating metalloproteinase-1 production in human dermal fibroblasts. FASEB J. 2006. 32. Keeling J, Herrera GA. The mesangium as a target for glomerulopathic light and heavy chains: pathogenic considerations in light and heavy chain-mediated glomerular damage. Contrib Nephrol. 2007. 33. Hideshima T, Ikeda H, Chauhan D, Okawa Y, Raje N, Podar K, et al. Bortezomib induces canonical nuclear factor-kappaB activation in multiple myeloma cells. Blood. 2009 34. Ludwig H, Drach J, Graf H, Lang A, Meran JG. Reversal of acute renal failure by bortezomib-based chemotherapy in patients with multiple myeloma. Haematologica. 2007 35. Minarik J, Scudla V, Tichy T, Pika T, Bacovsky J, Lochman P. Induction treatment of light chain deposition disease with bortezomib: rapid hematological response with persistence of renal involvement. Leuk Lymphoma. 2012 36. Lorenz EC, Gertz MA, Fervenza FC, Dispenzieri A, Lacy MQ, Hayman SR, et al. Long–term outcome of autologous stem cell transplantation in light chain deposition disease. Nephrology, Dialysis, Transplantation. 2008 37. Telio D, Shepherd J, Forrest D, Zypchen L, Barnett M, Nevill T. High-dose melphalan followed by auto-SCT has favorable safety and efficacy in selected patients with light chain deposition disease and light and heavy chain deposition disease. Bone Marrow Transplant. 2012 38. Royer B, Arnulf B, Martinez F, Roy L, Flageul B, Etienne I, et al. High dose chemotherapy in light chain or light and heavy chain deposition disease. Kidney Int. 2004 Feb 39. Masood A, Ehsan H, Iqbal Q, Salman A, Hashmi H. Treatment of Light Chain Deposition Disease: A Systematic Review. J Hematol. 2022 Aug 40. Weichman K, Dember LM, Prokaeva T, Wright DG, Quillen K, Rosenzweig M, et al. Clinical and molecular characteristics of patients with non-amyloid light chain deposition disorders, and outcome following treatment with high-dose melphalan and autologous stem cell transplantation. Bone Marrow Transplant. 2006 41. Fujita H, Hishizawa M, Sakamoto S, Kondo T, Kadowaki N, Ishikawa T, et al. Durable hematological response and improvement of nephrotic syndrome on thalidomide therapy in a patient with refractory light chain deposition disease. Int J Hematol. 2011 42. Kimura S, Ohkawara H, Ogawa K, Tanaka M, Sano T, Harada-Shirado K, Takahashi H, Ueda K, Shichishima-Nakamura A, Matsumoto H, Ikeda K, Kazama JJ, Hashimoto Y, Ikezoe T. Lenalidomide as a Beneficial Treatment Option for Renal Impairment Caused by Light Chain Deposition Disease. Intern Med. 2018 43. Mima A, Nagahara D, Tansho K. Successful treatment of nephrotic syndrome induced by lambda light chain deposition disease using lenalidomide: A case report and review of the literature. Clin Nephrol. 2018 44. Kobayashi A, Takeda A, Shinjo H, Iguchi D, Ito C, Okada E, Goto N, Futamura K, Okada M, Hiramitsu T, Narumi S, Watarai Y. Light Chain Deposition Disease Recurrence in Renal Allograft after Long-Term Remission. Nephron. 2023;147 Suppl 1:96–100. doi: https://doi.org/10.1159/000529776. Epub 2023 Feb 21. PMID: 36809757. 45. Leung N, Lager DJ, Gertz MA, Wilson K, Kanakiriya S, Fervenza FC. Long-term outcome of renal transplantation in light-chain deposition disease. Am J Kidney Dis. 2004 46. Short AK, O'Donoghue DJ, Riad HN, Short CD, Roberts IS. Recurrence of light chain nephropathy in a renal allograft. A case report and review of the literature. Am J Nephrol. 2001 47. Kaposztas Z, Kahan BD, Katz SM, Van Buren CT, Cherem L. Bortezomib successfully reverses early recurrence of light-chain deposition disease in a renal allograft: a case report. Transplant Proc. 2009 48. Hiyamuta H, Yamada S, Matsukuma Y, Tsuchimoto A, Nakano T, Taniguchi M, Masutani K, Yoshimoto G, Muta T, Akashi K, Kitazono T, Tsuruya K. Light Chain Deposition Disease in an Older Adult Patient Successfully Treated with Long-term Administration of Bortezomib, Melphalan and Prednisone. Intern Med. 2016;55(10):1319–25. doi: https://doi.org/10.2169/internalmedicine.55.5752. Epub 2016 May 15. PMID: 27181540. 49. Heilman RL, Velosa JA, Holley KE, Offord KP, Kyle RA. Long-term follow-up and response to chemotherapy in patients with light-chain deposition disease. Am J Kidney Dis. 1992 Jul;20(1):34–41. doi: https://doi.org/10.1016/s0272-6386(12)80,314–3. PMID: 1621676. 50. Kastritis E, Rousakis P, Kostopoulos IV, Gavriatopoulou M, Theodorakakou F, Fotiou D, Dialoupi I, et al. Consolidation with a short course of daratumumab in patients with AL amyloidosis or light chain deposition disease. Amyloid. 2021;28(4):259–266. 51. Milani P, Basset M, Curci P, Foli A, Rizzi R, Nuvolone M, et al. Daratumumab in light chain deposition disease: rapid and profound hematologic response preserves kidney function. Blood Adv. 2020.
